# Polyethylene Films Containing Plant Extracts in the Polymer Matrix as Antibacterial and Antiviral Materials

**DOI:** 10.3390/ijms222413438

**Published:** 2021-12-14

**Authors:** Magdalena Ordon, Magdalena Zdanowicz, Paweł Nawrotek, Xymena Stachurska, Małgorzata Mizielińska

**Affiliations:** 1 Center of Bioimmobilisation and Innovative Packaging Materials, Faculty of Food Sciences and Fisheries, West Pomeranian University of Technology in Szczecin, Janickiego 35, 71-270 Szczecin, Poland; magdalena.labuda@zut.edu.pl (M.O.); magdalena.zdanowicz@zut.edu.pl (M.Z.); 2 Center for Nanotechnology Research and Education, Department of Microbiology and Biotechnology, Faculty of Biotechnology and Animal Husbandry, West Pomeranian University of Technology in Szczecin, Piastów Avenue 45, 70-311 Szczecin, Poland; pawel.nawrotek@zut.edu.pl (P.N.); xymena.stachurska@zut.edu.pl (X.S.)

**Keywords:** SARS-Co-V2, Φ6 phage, antiviral properties, antibacterial properties, PE films, active foils, active films, plant extracts, CO_2_ extracts, raspberry seeds, pomegranate seeds, rosemary

## Abstract

Low density polyethylene (LDPE) films covered with active coatings containing mixtures of rosemary, raspberry, and pomegranate CO_2_ extracts were found to be active against selected bacterial strains that may extend the shelf life of food products. The coatings also offer antiviral activity, due to their influence on the activity of Φ6 bacteriophage, selected as a surrogate for SARS-CoV-2 particles. The mixture of these extracts could be incorporated into a polymer matrix to obtain a foil with antibacterial and antiviral properties. The initial goal of this work was to obtain active LDPE films containing a mixture of CO_2_ extracts of the aforementioned plants, incorporated into an LDPE matrix via an extrusion process. The second aim of this study was to demonstrate the antibacterial properties of the active films against Gram-positive and Gram-negative bacteria, and to determine the antiviral effect of the modified material on Φ6 bacteriophage. In addition, an analysis was made on the influence of the active mixture on the polymer physicochemical features, e.g., mechanical and thermal properties, as well as its color and transparency. The results of this research indicated that the LDPE film containing a mixture of raspberry, rosemary, and pomegranate CO_2_ extracts incorporated into an LDPE matrix inhibited the growth of *Staphylococcus aureus*. This film was also found to be active against *Bacillus subtilis*. This modified film did not inhibit the growth of *Escherichia coli* and *Pseudomonas syringae* cells; however, their number decreased significantly. The LDPE active film was also found to be active against Φ6 particles, meaning that the film had antiviral properties. The incorporation of the mixture of CO_2_ extracts into the polymer matrix affected its mechanical properties. It was observed that parameters describing mechanical properties decreased, although did not affect the transition of LDPE significantly. Additionally, the modified film exhibited barrier properties towards UV radiation. Modified PE/CO_2_ extracts films could be applied as a functional food packaging material with antibacterial and antiviral properties.

## 1. Introduction

Packaging for food products has been traditionally defined as a passive barrier that delays environment effects on food. However, trends in research have involved the development of packaging materials that could positively interact with food, and could play an active role in its preservation [1]. Packaging offering antimicrobial activity is a concept that may be defined as a version of active packaging in which the package, the food and the environment interact to extend the lag phase of microorganisms (responsible for food spoilage) and/or reduce their growth rate. Through this action, the shelf life of food products may be prolonged and their quality and safety can be better preserved [2,3,4,5,6]. An excellent example is the author’s [4] previous work, which demonstrated that boxes containing polyethylene (PE) films with coatings offering antimicrobial activity were found to be the most effective packaging that extended the freshness and quality of cod fillets after 144 h storage at 5 °C.

Active compounds/substances that are responsible for the antimicrobial activity of the packaging materials may be introduced into a coating or into polymer matrix [6]. Polymers are a very effective medium for active compounds, offering a possibility to incorporate different antimicrobial additives. An excellent example is low-density polyethylene (LDPE), which may incorporate nisin, organic acids, food preservatives, essential oils, natural plant extracts, or nanoparticles to obtain films offering antibacterial or antifungal activity [2,7,8,9,10]. Solano et al. [1] demonstrated that LDPE active films, which contained 1% and 4% (*w*/*w*) of thyme and oregano essential oils (Eos), incorporated into the polymer matrix showed antibacterial effect on *Escherichia coli* 0157:H7, *Salmonella* Typhimurium, and *Listeria monocytogenes*. Suppakul et al. [2] showed that LDPE containing plant extracts, such as clove extract, huanglian extract, or rhubarb extract were active against *E. coli*. The work of Lopez et al. [10] indicated that LDPE films with 4% fortified-cinnamon oil also showed antibacterial activity. These films inhibited *Bacillus cereus* and reduced the number of CFU’s of *Staphylococcus aureus* and *L. monocytogenes*. No effect was observed against Gram-negative bacteria. Many authors confirmed that active compounds introduced into the polymer matrix can lead to the creation of active packaging with antibacterial or antifungal properties, leading to an increase in the shelf-life of food products. Thus, one of the main interests in functional packaging is the inclusion of active compounds within polymeric matrices. Most of this interest is focused on additives obtained from natural sources, such as plant extracts or essential oils. Consumers perceive natural additives as low health risk compounds [11].

Supercritical extraction leads to the formation of highly active extracts which may inhibit the growth of Gram-positive, Gram-negative bacteria, yeast, and molds [6]. CO_2_ was found to be one of the most popular solvents, because carbon dioxide is cheap, non-toxic, readily available, and recognized as safe (GRAS) [12,13]. Additionally, this supercritical CO_2_ technology is an environmentally friendly technique [14]. The high antibacterial effect of supercritical extracts of selected plants, such as oregano, thyme, rosemary, hop, cloves, and basil on Gram-positive bacterial strains, e.g., *L. monocytogenes*, *B. subtilis*, and *S. aureus* was confirmed by Bhavya et al. [15].

Pomegranate fruits may be a source of polyphenolic compounds, their extracts can contain proanthocyanidins, anthocyanins, tannins, and lipophilic compounds, such as tocopherols and β-carotene, which have a wide range of health benefits. These substances can be active against *Pseudomonas aeruginosa*, *E. coli*, *B. cereus*, and *S. aureus* [16,17]. Rosemary plant extracts were also found to be highly active against microorganisms. Their effect on mold and bacterial strains, such as *Aspergillus parasiticus*, *L. monocytogenes*, or *S. aureus* was observed by several authors [18,19,20]. The antimicrobial properties of these plant extracts can be attributed to phenol diterpene, rosmarinic acid, carnosic acid, carnosol, rosmanol, ursolic acid, and betulinic acid [18,19,20]. Raspberry extracts are also very rich in microbiologically active compounds, and can contain polyphenols, anthocyanins, flavanols, hydroxybenzoic acids, hydroxycinnamic acids, and hydroxybenzoic acids. As was mentioned in a previous study [6], the most common antibacterial action mechanisms of these agents are those related to a bacteriolytic type, associated with mechanism based on the disruption of the bacterial plasma membrane or on efflux pump inhibition. Many authors confirmed that active substances obtained from raspberries demonstrated antifungal, antibacterial, and antiviral properties [6,21].

Among the worst pandemics recorded during the twentieth century was the flu of 1918–1920. Severe acute respiratory syndrome coronavirus (SARS-CoV-2), known as COVID-19, has emerged as a major threat to human existence in the twenty first century [22]. Thus, the entire human population is being threatened with exposure to this highly contagious virus [23]. SARS-CoV-2 enters the host cell by attaching S glycoproteins to angiotensin-converting enzyme 2 (ACE2) that is mediated via serine protease TMPRSS211. The possible candidate drugs should be based on the inhibition of binding ACE2 to SARS-CoV-2 via the inhibition of ACE2 and TMPRSS211 or polyclonal antibody against S glycoproteins. The possible drug may also inhibit the replication of a coronavirus [22]. The possible antiviral material, e.g., packaging, active material should prevent docking of the virus to host cells. OH groups, which can be found in flavonoids, tannins, or rosmarinic acid from CO_2_ plant extracts, may form hydrogen and ion bonds containing outer groups of proteins, e.g., positively charged amino groups. The polyphenolic compounds bind to viral proteins in the envelope, that often prevents docking of the virus particles to the host cells [6]. The global spread of the pandemic caused by the SARS-CoV-2 is a problem that poses unprecedented risks to human/consumer health. To prevent viral transmission, the use of synthetic polymers for customers by retailers has become essential. It should be also mentioned that it is not possible to disinfect every single piece of food packaging or area after being touched by consumers in supermarkets. That is why the use of single-use food packaging and plastic bags to carry groceries increased during the COVID-19 pandemic [5,6]. In order to ensure customer safety, supermarkets could also develop additional health safety measures, such as active polymeric packaging with antiviral activity. This safe packaging has to have to be modified with active additives to parallelly protect food products by an internal layer and to protect customers with an external layer of the packaging material. Therefore, multilayer polymer laminates with internal layer with antibacterial activity and external layer with antiviral effect may be a solution. From an ecological point of view, multilayers might not be the best solution, due to difficulties in recycling, thus monomaterial laminates, e.g., a polyethylene film containing different, natural, active substances in each layer can be proposed. A monolayer material, e.g., PE film with active agents incorporated into their matrix may also be a solution; however, these additives should exhibit wide activity spectrum, e.g., both antibacterial and antiviral [5,6,24,25]. Active compounds from raspberry, rosemary, and pomegranate fruit extracts, were confirmed to be active against viruses [18,25]. Rosemary extracts inhibited SARS-CoV-2 particles [18]. Raspberry and pomegranate extracts were effective against the Dengue virus and hepatitis A and B. Kaempferol as active compound from berry fruits was found to have an effect on coronaviruses [25].

The previous study [6] demonstrated that PE films covered with active coatings based on methyl-hydroxy-propyl-cellulose containing mixtures of raspberry, rosemary, and pomegranate fruit extracts were confirmed as antibacterial coatings that could extend the shelf life of food products. All of the analyzed coatings (containing mixtures of extracts) also had antiviral activity, due to the influence the coatings/layers on the activity of Φ6 bacteriophage, selected as a surrogate for SARS-CoV-2 particles. It can be concluded that coatings obtained in the previous study would also be active against coronavirus particles. Additionally, the synergism of active compounds from analyzed CO_2_ extracts was also observed. The coatings containing the extract mixtures were found to be more active against bacterial strains than the coatings containing pure extracts. The most effective results were obtained with the mixture of all three extracts (a ratio of 1:1:1). This is why the mixture of raspberry, rosemary, and pomegranate fruit extracts could be incorporated into a polymer matrix to obtain films with antibacterial and antiviral properties.

LDPE is one of the most popular and the cheapest polymers used in packaging manufacture. It is characterized by good mechanical and barrier against moisture, and it is easily processable and thermoformable. 

The initial purpose of the study was to obtain active, LDPE films containing the mixture of raspberry, rosemary, and pomegranate CO_2_ extracts in the matrix. The second aim of presented study was to determine the antibacterial properties of active films against Gram-positive and Gram-negative bacterial strains. The goal was also to determine the antiviral effect of the film on Φ6 bacteriophage (used as an eucaryotic virus surrogate). Based on the results of the authors’ previous work [5,6], an analysis of the antiviral activity of LDPE active films was carried out using Φ6 bacteriophage that represented a good surrogate for testing airborne viruses, such as coronaviruses [5,6,26]. Additionally, the impact of the active mixture on the polymer matrix properties, i.e., color, mechanical, and thermal properties, FTIR and UV-Vis spectroscopy, as well as color, were also investigated.

## 2. Results

### 2.1. Antibacterial Analysis

**Figure 1:**
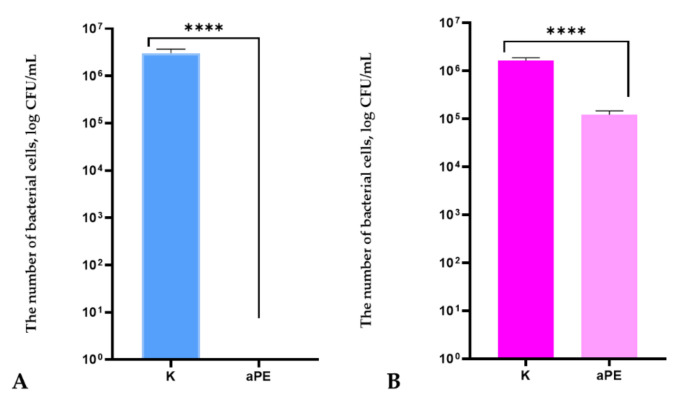
The influence of active polyethylene film (aPE) on Gram-positive bacteria growth **A**: *S*. *aureus*; **B**: *B. subtilis*. K—PE film; aPE—PE film containing a mixture of CO_2_ extracts of raspberry seeds, pomegranate seeds, and rosemary in a polymer matrix. Unpaired *t*-test: ****—*p* < 0.0001.

**Figure 2:**
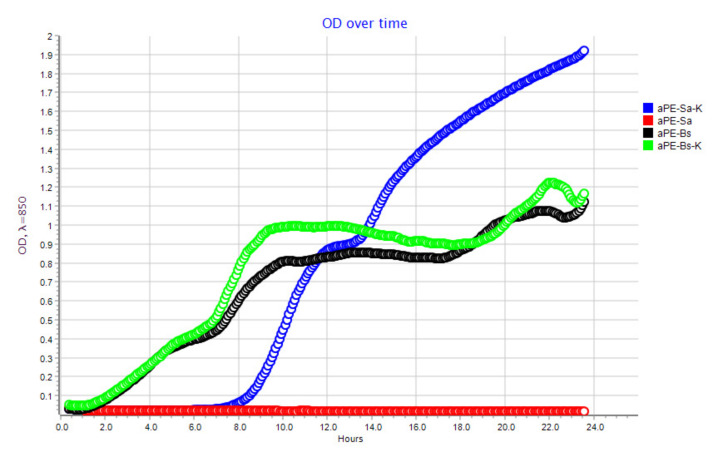
OD over time for *S*. *aureus* (Sa) and *B. subtilis* (Bs) after their incubation with the aPE; K—PE film; aPE—PE film containing a mixture of CO_2_ extracts of raspberry seeds, pomegranate seeds, and rosemary in polymer matrix.

The results of this study indicated that LDPE films containing the extracts (aPE) inhibited the growth of *S*. *aureus* cells (Figure 1A), which meant that the films exhibited bactericidal effect on the microorganism cells. An analysis of the growth rate of *S. aureus* in real time confirmed the antibacterial effect of the active film on the bacterial strain (Figure 2). Analyzing the OD over time for the LDPE film without active compounds (K), an increase in OD was observed, which meant that the microorganism cells were viable (Figure 2). The OD curve was constant for the bacterial culture after its incubation with aPE, and an increase in OD was not observed. The test confirmed that *S. aureus* cells growth was inhibited by the aPE.

The results of this research showed that LDPE films obtained through the incorporation of extracts were also found to be active against *B. subtilis* (Figure 1B). These films did not inhibit the bacterial strain, but decreased the number of microorganism cells from 1.63 × 10^6^ to 1.22 × 10^5^ (CFU/mL). The differences between the numbers of viable cells were not high but they were found to be significant, as confirmed by a one-way ANOVA test (*p* < 0.0001). To confirm the antibacterial effect of films on *B. subtilis*, an analysis of its growth rate over time was carried out. The results of the test showed that OD over time for *B. subtilis* after its incubation with active films was lower than the OD over time for the strain incubated with a control sample (LDPE film devoid of active compounds in the polymer matrix). It was also observed that OD over time increased significantly after 22 h in the case of *B. subtilis* incubated with active PE film (Figure 2). To summarize, LDPE containing a mixture of analyzed plant extracts in a polymer matrix exhibited a bacteriolytic effect on *S. aureus* and a low antibacterial activity against *B. subtilis* strains.

**Figure 3:**
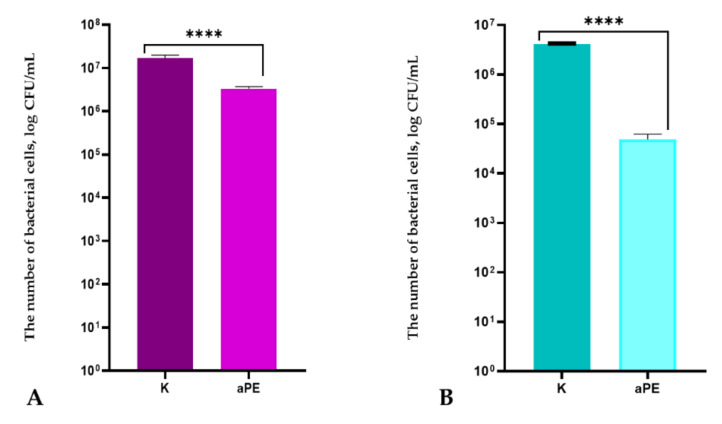
The influence of active polyethylene film on Gram-negative bacteria growth **A**: *E*. *coli*; **B**: *P. syringae*. K—PE film; aPE—PE film containing a mixture of CO_2_ extracts of raspberry seeds, pomegranate seeds, and rosemary in polymer matrix; unpaired *t*-test: ****—*p* < 0.0001.

**Figure 4:**
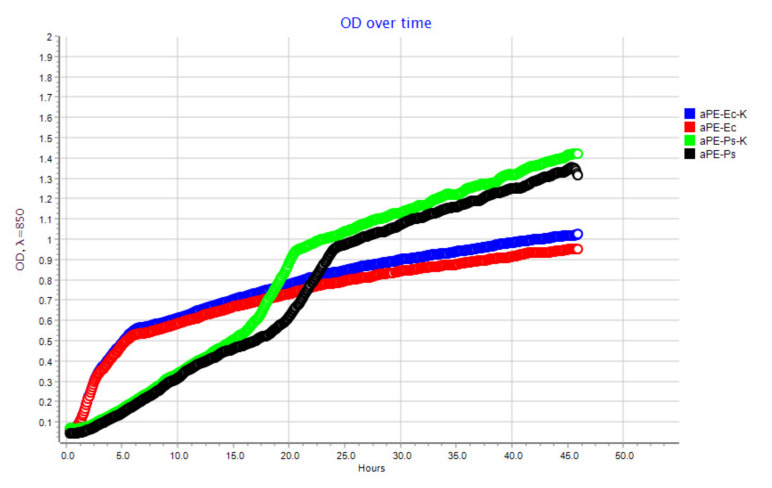
OD over time for *E*. *coli* and *P. syringae* after their incubation with the aPE; K—PE film; aPE—PE film containing a mixture of CO_2_ extracts of raspberry seeds, pomegranate seeds, and rosemary in polymer matrix.

It was demonstrated that LDPE foil containing the mixture of CO_2_ extracts as a source of active compounds did not inhibit the growth of *E. coli* and *P. syringae*, but decreased the number of microorganism cells. As seen below (Figure 3A), the number of *E. coli* cells decreased slightly from 1.67 × 10^7^ to 3.34 × 10^6^ (CFU/mL). Statistical analysis indicated that the differences between the numbers of *E. coli* cells were significant (*p* < 0.0001). It was found that on analyzing the antibacterial effect of active LDPE film on the *P. syringae* strain, statistically significant, but not high, antibacterial activity was noted. The number of *P. syringae* cells were seen to decrease from 4.11 × 10^6^ to 4.88 × 10^4^ (CFU/mL) (Figure 3B). Comparing the activity of aPE, it was found that seen to have higher antibacterial effect on *P. syringae* than on *E. coli*. To confirm the antibacterial activity of LDPE foil with active compounds against *E. coli*, an analysis of its growth rate over time was carried out. The results of the experiment showed that OD over time for this bacterial strain after incubation with active foil sample was lower than the OD over time for the strain which was incubated with a control sample (LDPE film devoid of active substances in the polymer matrix). The active LDPE foil samples (containing the mixture of analyzed plant CO_2_ extracts in the polymer matrix) were confirmed to have an antibacterial effect on *E. coli* and antibacterial activity against *P. syringae* strains. As noted below (Figure 4), a fall in the OD curve for *P. syringae* after 44 h was seen. A fall in OD was not observed for *E. coli*, which meant that the active films had higher antibacterial effect on *P. syringae* than on *E. coli*.

### 2.2. Antiviral Analysis

**Figure 5:**
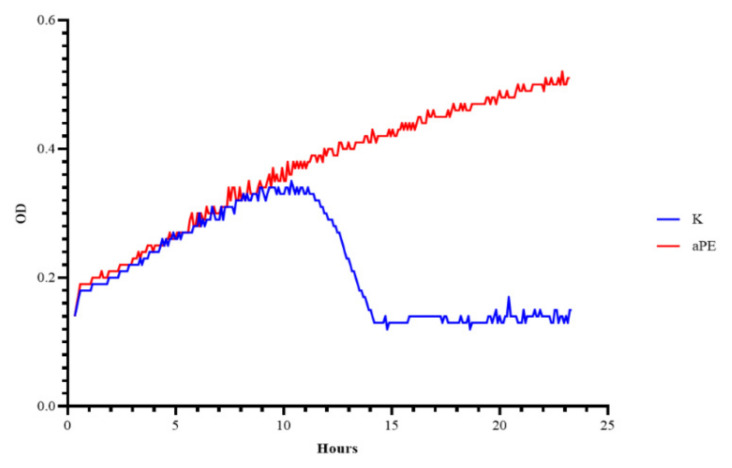
OD over time for *P. syringe* incubated with Φ6 phages after their incubation with the aPE (phages were added when OD = 0.2, amount of phage MOI = 1). K—PE film; aPE—PE film containing a mixture of CO_2_ extracts of raspberry seeds, pomegranate seeds, and rosemary in polymer matrix.

**Figure 6:**
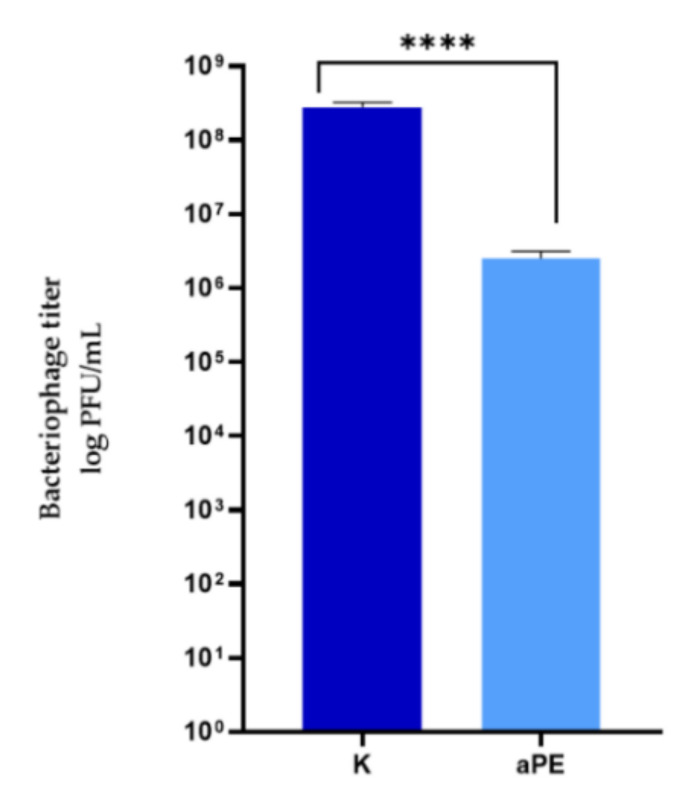
The influence of active polyethylene film on bacteriophage titer. K—PE film; aPE—PE film containing a mixture of CO_2_ extracts of raspberry seeds, pomegranate seeds, and rosemary in polymer matrix. unpaired *t*-test: ****—*p* < 0.0001.

An analysis of the growth rate in real time, the OD of the *P. syringae* cells over time, cultivated with Φ6 phages after their incubation with active LDPE films was carried out to demonstrate the antiviral effect of the films with the mixture of CO_2_ extracts of raspberry, rosemary, and pomegranate fruit incorporated into a polymer matrix. Figure 5 demonstrates the OD over time of the bacterial host incubation at 28 °C. An analysis of the OD over time of the microorganism culture cultivated with the Φ6 phages (after their incubation with the LDPE film as control sample (K)) was performed and an OD fall was observed after 12 h of phage incubation with the *P. syringae* strain, which confirmed that Φ6 particles eliminated most of the host cells. The results show that K sample (LDPE without extracts) was not effective against ph6 phages. The results of the experiments, performed according to a modified ISO 22196-2011 standard, demonstrated that the incubation of phage Φ6 with active LDPE films did not inactivate the bacterial virus particles completely, but decreased their number (Figure 6). A statistical analysis indicated that the differences between numbers of Φ6 particles were significant (*p* < 0.0001).

### 2.3. FTIR Analysis

**Figure 7:**
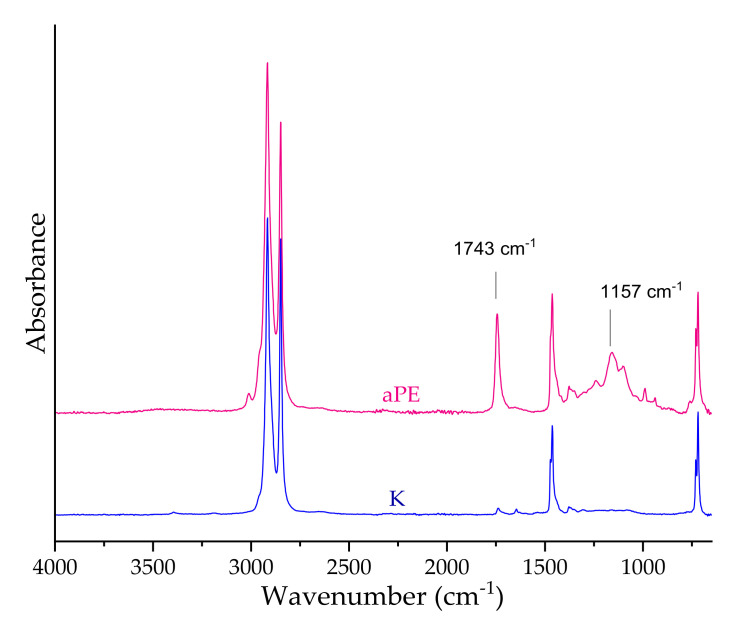
The images of LDPE films. K (blue)—PE control film; aPE (pink)—PE film containing a mixture of CO_2_ extracts of raspberry seeds, pomegranate seeds, and rosemary in polymer matrix.

The results of this research indicated that there were six regions viewed in the FT-IR spectra, extending over (1) ranges from 3200 to 2800 cm^−1^; (2) ranges from 1800 to 1600 cm^−1^; (3) ranges from 1500 to 1400 cm^−1^; (4) ranges from 1400 to 1000 cm^−1^; (5); and (6) ranges from 800 to 650 cm^−1^. In the case of 2916 and 2848 cm^−1^ peaks, peaks, stimulated by aliphatic C-H stretching bonds were noted. Additionally, a spectrum peak at 1462 cm^−1^ can be observed for a peak with bending C-H bond absorption. These peaks confirmed that the foil sample was polyethylene. Alternatively, a spectrum peak at 1743 cm^−1^ was observed for a peak with stretching C=O bonds induced absorption; 1372 cm^−1^; 1240 cm^−1^ peaks induced by asymmetric O-H bonds induced absorption and peaks at, 1157 and 1101 cm^−1^ (C-O stretching bonds vibrations) corresponded to flavanols, flavanone, chloro-flavones, ursolic acid, and tannins. It was also noted that the absorption peaks were assigned to flavones. These peaks were attributed to the characteristic vibrations of active compounds from a mixture of CO_2_ extracts of raspberry, rosemary, and pomegranate fruit. FTIR spectrum analysis confirmed that the analyzed LDPE films contained active substances in the polymer matrix (Figure 7). Comparing K and aPE, there is no peak shifts, which means that chemical reaction between PE and the additive did not occur and interaction has a physical character.

### 2.4. SEM Analysis

**Figure 8:**
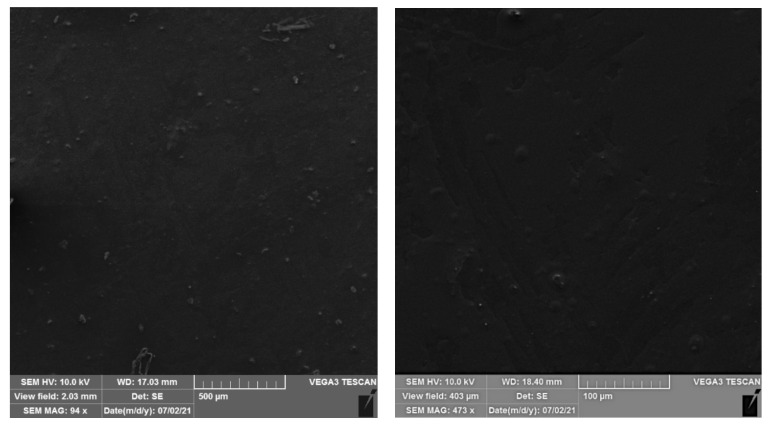
The SEM images of aPE films. K—PE film; aPE—PE film containing a mixture of CO_2_ extracts of raspberry seeds, pomegranate seeds, and rosemary in polymer matrix.

Figure 8 shows micrographs of aPE films. SEM analysis demonstrated that the thin layer of extracts mixture was observed on the surface of PE film. The surface was quite smooth, without holes but some knobs can be observed. The SEM analysis confirmed the migration of extracts from the polymer bulk. These observations correspond with the FTIR analysis results (presence of active compounds from the extract on PE surface) and tensile tests (phases separation).

### 2.5. Mechanical Properties Analysis

Mechanical test results are listed in Table 1. It can be seen that the presence of the extract in the polymer matrix affected its mechanical properties. All parameters decreased: TS and EB ca. 25% and YM ca. 13% in comparison to K sample.

### 2.6. Colour Determination

The color determination of the reference sample and aPE is presented in Table 1. Due to the orange-brownish color of the active mixture, the modified PE material had a slightly yellow hue, shown by a high b* value (2.80), it also seems that sample has a green hue (a* −0.28); however, this was not visible by the naked eye. The mixture presence did not have an effect on the lightness of the film (L* value). Visually, the difference was less noticeable than, e.g., between PP and PP films with a mixture of essential oils [27].

### 2.7. UV-Vis Spectrophotometry

**Figure 9:**
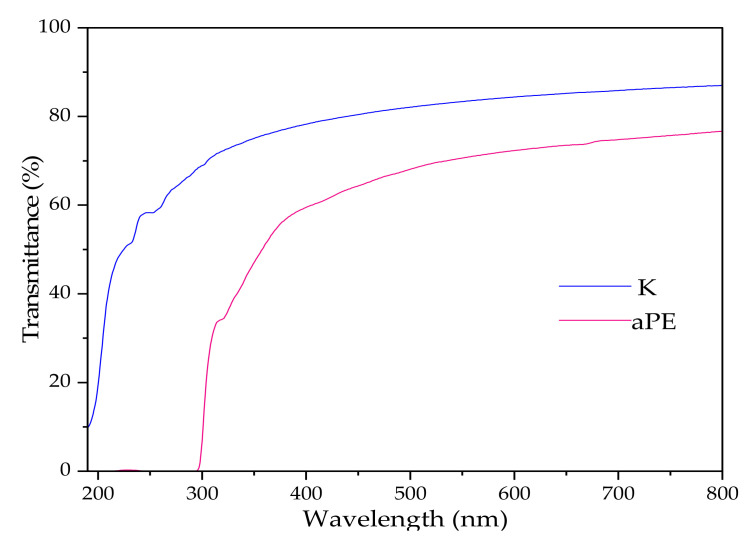
Transmittance curves at UV-Vis range of K and aPE samples.

Figure 9 shows film transmittance. At 650 nm transmittance is 85% and 74% for K and aPE, respectively. Despite this, the aPE exhibited lower parameter value than the reference sample, and was found to still be highly transparent. It is noteworthy that the aPE film is not transparent in the case of UV radiation (there is high absorbance peak at 190–300 nm, though the results are not shown). The barrier properties against UV light are the result of a presence of benzyl rings from the rosemary oil, an ellagic acid from the pomegranate extract in the active mixture.

### 2.8. Thermal Characterization

**Figure 10:**
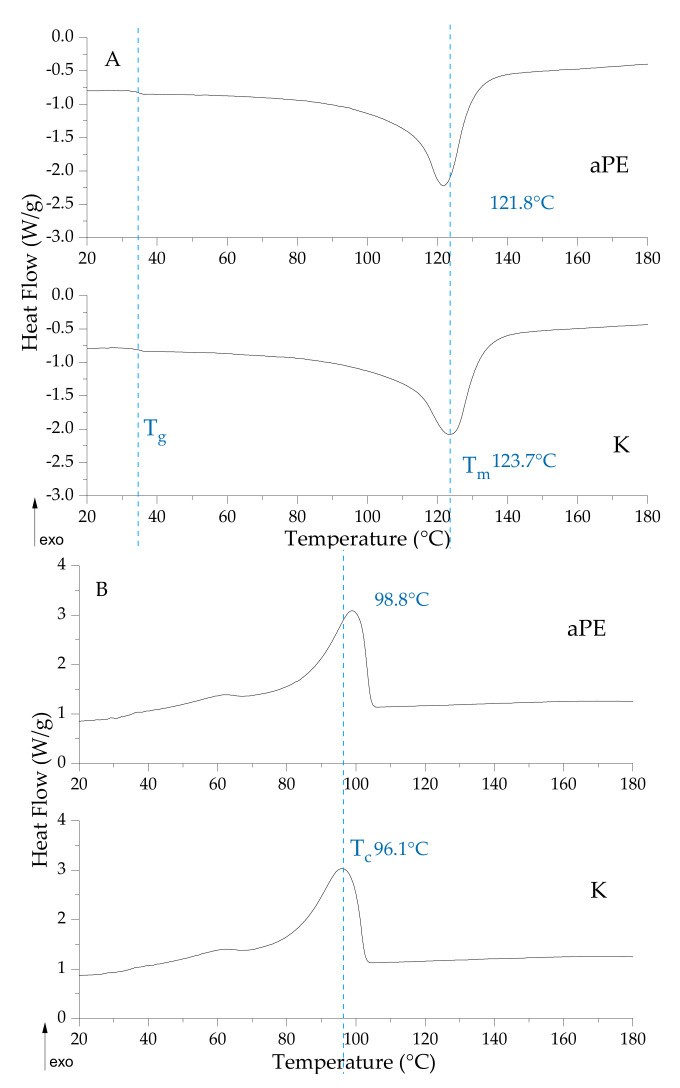
DSC curves of the films in heating (**A**) and cooling (**B**) scan; T_g_—glass transition, T_m_—melting transition, T_c_—crystallization transition.

Figure 10 shows the phase transition of the reference sample and modified active polymer. As seen here, the active mixture did not affect the glass transition of the polyethylene; however, there are slight, insignificant differences in melting and crystallization temperature values. There is also no difference in ∆H values, a result of the degree of polymer crystallinity.

**Figure 11:**
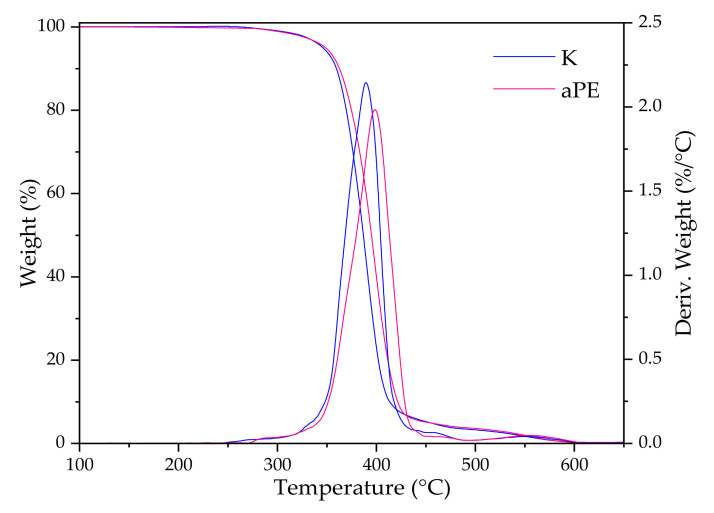
TG and DTG curves of K and PE samples.

TG curves in Figure 11 show that there is no significant difference in thermal stability (the onset decomposition temperature for both samples is nearly 329 °C); however, in the case of the DTG curves, there is a noticeable difference in maximum peak: 389 °C for K and 398 °C for aPE, respectively. The modified PE is slightly more thermally stable that the neat K sample.

## 3. Discussion

The addition of the active extracts mixture to the LDPE matrix facilitated processing via extrusion (5 wt% in PE). The mixture acted as lubricant and plasticizer for the polymer and led to a decrease in the processing temperature (standard minimal temperature for PE extrusion was 180 °C and aPE was 160 °C). Films obtained directly from the cast extruder die were rather yellowish and highly transparent (see Supplementary Figure S1), however after few days of storage they became slightly opaque.

As the result of the previous study [6], the synergistic effect between CO_2_ extracts of raspberry, rosemary, and pomegranate seeds in the activity of the coatings on polyethylene was noted. The results of this work indicated that 24 h incubation of active LDPE films (containing the extract) with *S*. *aureus* strain inhibited the growth of bacterial cells. It may be concluded that the active compounds from the mixture of extracts, such as carnasoic acid, carnosol, and phenol diterpenes [18,19] could be the cause of the bacteriolytic effect of aPE on the growth of the bacterium. It is most likely that these substances inhibited efflux pump or increased permeability in the bacterial cell wall [28]. In the case of the other Gram-positive strain: *B. subtilis*, a low (1 log), antibacterial effect of the aPE on the strain was observed. Similarly, a 1 log decrease in the amount of *B. subtilis* cells was noted in this study and a previous paper [6]. The diterpenes from the extracts could cause a disturbance in protein and DNA metabolism which could then lead to the antibacterial effect of active films.

The results of this research showed that the aPE film was also found to be active against *E. coli* and *P. syringae*. Analyzing the antibacterial effect of the active LDPE film on the *E. coli* and *P. syringae* strains, resulted in low but statistically significant antibacterial activity being seen. Comparing the activity of the LDPE film containing the mixture of extracts in the matrix, it was clear that the aPE films had higher antibacterial effect on *P. syringae* (2 log) than on *E. coli* (1 log). Similar results were obtained in previous research [6]. Comparing the results of the current work to the mentioned study, it is important to note that the active coatings containing CO_2_ extracts exhibited a bacteriolytic effect on *P. syringae*. The active films with the mixture of the same extracts incorporated int polymer matrix exhibited an antibacterial effect on *P. syringae*. The reason for the higher activity of coatings over the activity of the films could be caused by the concentration of active compounds on the surface of the polyethylene film. It could be assumed that the migration of active compounds from the coatings was higher than from the polymer bulk.

The results of this research showed that the aPE films were active against Φ6 particles. This was confirmed by the results from previous studies carried out by the authors [5,6], which demonstrated that active coatings with the addition of the same extracts or active coatings that contained ZnO nanoparticles, geraniol, and carvacrol inactivated Φ6 bacteriophage particles. It could be suggested that the active coatings and active films would be also effective against eukaryotic SARS-CoV-2. Pan et al., and Prussin II et al., observed that phages may be used as surrogates for eukaryotic viruses, such as coronaviruses [26,29]. These assumptions were also confirmed by Surucic et al., [30] who demonstrated that pomegranate polyphenolic substances have the potential to attenuate the ability of SARS-CoV-2 S-glycoprotein to bind to the ACE2 receptor and therefore be excellent candidates for possible therapeutic application.

The SEM analysis demonstrated that the samples with 5% addition of CO_2_ extracts (aPE) had partially smooth with some small knobs however, holes were not observed. It indicates, that extracts migrated without destructive effect on PE matrix, as can be observed when some additives evaporate [31]. The thin layer formed with extracts mixture was observed on the aPE surface, confirming migration of the additive. The morphology of polyethylene films containing active compounds incorporated into polymer matrix were also observed by Pillai et al. [31]. The authors demonstrated that the homogenous morphology of LDPE films was only observed for films which contained a 1% addition of thyme oil. The authors noted that the surface of the films changed with the addition of higher amounts of thyme oil. The film containing a 2.5 and 5% addition of thyme oil had rougher surface than the films containing only 1% of thyme oil. The surface of the films with higher concentrations of thyme oil showed crater-like indentations on their surfaces. The authors concluded that the change in morphology could be due to the partial vaporization of essential oil from the polymer matrix during the extrusion process. To summarize, the results of the SEM analysis may lead to the conclusion that the mixture of active extracts did not evaporate from the LDPE matrix.

The FTIR analysis showed the presence of a 2916; 2848 cm^−1^ and 1462 cm^−1^ peaks proving that that the sample that was analyzed was polyethylene. The obtained results were confirmed by Torres et al., [11]. Alternatively, spectrum peaks at 1743 cm^−1^; 1372 cm^−1^; 1240 cm^−1^; 1157 cm^−1^ and 1101 cm^−1^ were observed. These peaks corresponded to flavanols, flavanone, chloro-flavones, ursolic acid, tannins, and flavones, respectively. These peaks were attributed to the characteristic vibrations of active compounds from a mixture of CO_2_ extracts of raspberry, rosemary and pomegranate fruit. The FTIR spectrum analysis confirmed that the analyzed LDPE films contained active substances in the polymer matrix. The results revealing extracts migration on the PE surface corresponded with the SEM analysis results (presence of the additive on PE surface) and tensile tests (phases separation). A similar analysis was performed by other authors who have also confirmed the presence of active compounds in the PE matrix [9,11,31]. The explicit appearance of peaks in the aPE spectra suggest the presence of a modifying mixture on the polymer surface. This corresponds to the antimicrobial tests that were made, where the migration of the extracts lead to their increased availability for contact with the pathogens.

The results of the study demonstrated that an active mixture of CO_2_ extracts did not affect the glass transition of the polyethylene; however, slight but not significant differences in melting and crystallization temperature values were observed. There was also no difference in ∆H values, assigned to the degree of polymer crystallinity. Similar results were obtained by Pillai [31], where PE with PE/5% thyme oil were compared. The lack of any impact of the active mixture on the polymer matrix could be related to the poor miscibility of the matrix and modifier. Thanks to this behavior the active mixture migrated onto the polymer surface leading to the contact of active compounds of the extracts with microorganisms, causing a bacteriolytic or antibacterial effect on the selected bacterial strains and the elimination of the coronavirus particles.

The results of the work showed that there were no significant differences in the thermal stability of polyethylene after the incorporation of the extracts into a polymer matrix. Similar results were obtained by Pillai et al. [31] for PE with thyme oil in low concentration (up to 2.5 wt% as well as in Dong’s et al. work for LDPE with 1% of rosemary or rosemary and cinnamon oils [32]. DSC and TGA results suggest that aPE can be processed with similar parameters as unmodified PE, this is because there is a small visible difference between both materials.

The mechanical tests demonstrated that the incorporation of an active mixture of CO_2_ extracts into the polymer matrix affected its tensile properties and all parameters decreased. This can be explained by the matrix inhomogeneity and separation of phases created by the presence of small molecular sized active compounds at high concentration in the polymer bulk and their lower hydrophobic character compared to PE. However, the difference was found to not be significant when considering the application as protective, mainly single-use materials. Similar results were also obtained in other studies. Pillai et al., [31] prepared and described LDPE films with the addition of thyme oil. The addition of thyme oil to the LDPE matrix resulted in slight modifications of the tensile properties of the films that were obtained via blow extrusion. The 20% decrease in Young’s modulus was observed for the thermocompressed film containing 5 wt% of essential oil than compared to the reference material. In Canales et al. study [9] a reduction in tensile properties after the introduction of carvacrol or trans-cinnamaldehyde was also observed. PE as highly hydrophobic material is not effectively miscible with semi-hydrophilic extract components, leading the migration of the active additive from the polymer bulk to the surface. This phenomenon seems to be a disadvantage from a mechanical point of view, however, the weak bonding of the additive to the matrix is not unexpected, due to the increase in the availability of the active mixture for pathogens. Moreover, due to the migration from bulk to the surface, the release of the active substance is extended over time. As a packaging material it can be an advantage over coatings, which can be easily removed from the material by consumers and store staff, whereas modifiers introduced into polymer bulk migrates little by little and even if a small amount of the active substance is removed, some part can be released again after some time.

UV radiation may contribute to a deterioration in the physico-mechanical, optical, and antibacterial activity of polymer-based packaging materials. The introduction of active compounds that are not resistant to UV irradiation into the LDPE matrix could decrease packaging effectiveness in the case of UV-radiation, while the addition of active substances into the polymer matrix not sensitive to UV-aging, or the addition of an antibacterial with shielding properties, may prevent this decrease. The active LDPE film created in this research was not found to be UV radiation transparent. The barrier properties towards UV light are related to the presence of benzyl rings from rosemary oil and ellagic acid from the pomegranate extract in the active mixture, incorporated into the polymer matrix. These properties are important in the case of prolonged application and can prevent or reduce material photoaging. The incorporation of antibacterial additives into the PE matrix with shielding properties was analyzed by the authors in a previous study [8]. It was found that ZnO nanoparticles (as substances with shielding properties) prevented the active film from losing its antibacterial activity after uv-aging tests.

## 4. Materials and Methods

### 4.1. Materials

The test microorganisms used in the experiments were purchased from the Leibniz Institute DSM (Deutsche Sammlung von Mikroorganismen und Zellkulturen). They were *B. subtilis* DSM 1090, *S*. *aureus* DSM 346, *E*. *coli* DSM 498, and *P*. *syringae* van Hall 1902 DSM 21482. Phage Φ6 DSM 21518 (a segmented double-stranded RNA phage of the *Cystoviridae* family) was used as a SARS-CoV-2 surrogate.

Low-density polyethylene (LDPE) (410E, Dow Inc., Midland, Michigan) was used to obtain active films. ATMER 110-LQ-(CQ) (CRODA, Chocques, France) was used to incorporate the active substances into the LDPE matrix. Ecological CO_2_ extracts (ECOSPA, Józefosław, Poland) of: a. raspberry seeds, b. rosemary, c. pomegranate seeds were used as active additives. To determine the antibacterial and antiviral activity of any LDPE films, TSA, TSB, and Luria-Bertani (LB) broth (Merck, Darmstadt, Germany) were used. All mediums were prepared according to the manufacturer’s instructions.

### 4.2. PE Films Preparation

Before PE regranulation, the extracted mixture (5 wt% in PE) was stirred with ATMER (1 pph per PE, used as plasticizer and compatibilizer) by magnetic stirrer for 10 min. Then, PE pellets were mixed with the liquid system by hand. The material was extruded with a twin-screw extruder (L/D = 40, ten heating zones, Labtech Engineering, Thailand) The thermal profile was 145/160 × 8/155 °C. The final modified PE pellets were extruded through a flat die using Chill-Roll Cast Film Extrusion Line, Type LCR-300 Co-Ex (Labtech Engineering, Thailand) to obtain films with a thickness of 180 to 220 µm. The following temperature profiles during the tests were as follows: extruder: 145/155/160/160, pipe, extruder and die 160 °C, then the PE film coil was stored in a PE bag in ambient conditions. The modified PE has aPE acronym and PE without extracts is named K.

### 4.3. Antibacterial Analysis

The LDPE foil samples without active substances (control samples—K) and LDPE foil samples containing a mixture of extracts acting as active agents were cut into square shapes (3 cm × 3 cm) at 22 °C and tested.

The antibacterial effectiveness of the obtained active LDPE foil samples against Gram-negative *P. syringae*, *E. coli*, and Gram-positive *B. subtilis* and *S. aureus* strains, compared to the LDPE films devoid of active compounds was carried out according to the ASTM E 2180-01 standard [33].

To confirm the antibacterial activity of the aPE against bacterial strain growth rate in real time, after their incubation with the active film squares, a modified ISO 22196-2011 standard was used [5,6,33]. The LB broth was introduced into Biosan bioreactors (BS-010160-A04, Biosan, Riga, Latvia). The overnight bacterial culture was then added (each strain separately) to 30 mL of LB broth and incubated for 24 h at 28 °C. Four tests were carried out simultaneously and it was possible to analyze the antibacterial effect of aPE film on *B. subtilis* and on *S. aureus* strains during the experiment. The activity of aPE film against *E. coli* and *P. syringae* was analyzed in the next experiment according to the method described above.

### 4.4. Antiviral Analysis

As a first step of the research, phage Φ6 particles were purified according to Bhetwal et al. [34]. The obtained lysate was then prepared according to Bonilla et al. [35]. The antiviral activity of the aPE was compared to the LDPE films devoid of active compounds in a polymer matrix and were performed according to a modified ISO 22196-2011 standard [36]. Finally, Φ6 particle amplification was carried out using the Skaradzińska et al. method [37].

To analyze the influence of Φ6 particles on *P. syringae* growth rate in real time, Φ6 lysate was incubated with K and with aPE (separately) according to the ISO 22196-2011 standard [36]. The LB broth was introduced into two BioSan bioreactors (BS-010160-A04, BioSan, Riga, Latvia). Next a host overnight culture was added to 30 mL of LB broth and incubated at 28° until OD = 0.2 (optical density). Two Φ6 bacteriophage lysates were amplified in respective *P. syringae* (one lysate—after incubation with the PE, one lysate—after incubation with the aPE). Next, 11 µL of Φ6 lysate (MOI = 1, 1 phage per 1 bacterial cell) were added to a host culture (OD = 0.2) and incubated for 24 h at 28 °C.

### 4.5. SEM

aPE film was analyzed using a scanning electron microscope (SEM). The microscopic analysis was performed using a Vega 3 LMU microscope (Tescan, Brno-Kohoutovice, Czech Republic). The experiments were necessary to demonstrate if the active film was homogeneous or not. An analysis was performed at room temperature with a tungsten filament and an accelerating voltage of 10 kV was used to capture SEM images. All specimens were viewed from above.

### 4.6. FT-IR

A Fourier transform infrared (FT-IR) spectra of the K and aPE films were measured using FT-IR spectroscopy (Perkin Elmer Spectrophotometer, Spectrum 100, Waltham, MA, USA), operated at a resolution of 4 cm^−1^, over four scans. Samples were cut into square shapes (1 cm × 1 cm) and placed directly on the ray-exposing stage. The spectra were recorded at a wavelength of 650–4000 cm^−1^.

### 4.7. Mechanical Analysis

Mechanical tests for PE and aPE films were performed using Zwick/Roell Z 2.5 (Zwick, Wrocław, Poland) testing machine (load cell 2.5 kN). The films (a thickness of 0.20–0.25 mm) were cut into 12 mm wide stripes. The initial grip separation was 50 mm and the cross head speed was 100 mm/min. At least six replicated samples for each system were tested and the mechanical parameters: elongation at break (EB), tensile strength (TS), and Young’s modulus (YM) with standard deviations were calculated with TestExpert software.

### 4.8. UV-Vis Spectroscopy and Colour Determination of the Films

The UV–Vis spectra analysis (transmittance and absorbance in a region of 200–800 nm) of the samples was conducted using UV–Vis Thermo Scientific Evolution 220 spectrophotometer (Waltham, MA, USA). Color determination was performed with a Colorimeter (CR-5, Konica Minolta, Tokyo, Japan) with CIELab color scale. Samples were analyzed in 5 repetitions.

### 4.9. DSC Analysis of the Films

Differential scanning calorimetry (DSC) analysis was performed with Q100 DSC (TA Instruments Inc., New Castle, DE, USA). The samples (reference PE bead and aPE films) were analyzed in a nitrogen atmosphere, in a heating–cooling–heating cycle. The temperature range cycles were as follows: first heating from 0 to 150 °C, cooling from 150 to −70 °C and second heating from −70 to 350 °C with a heating/cooling rate of 10 °C/min.

Thermogravimetric analysis—TGA (Q2500, TA Instruments Inc., New Castle, Delaware, DE, USA) for the films was performed using a platinum pan under 25 mL/min air flow, in a temperature range of 40–900 °C at a heating rate 10 °C/min.

### 4.10. Statistical Analysis

Statistical significance was determined using a variance analysis (One-way ANOVA). The values were considered significantly different when *p* < 0.05. All analyses were performed with GraphPad Prism 8 (GraphPad Software, San Diego, CA, USA).

## 5. Conclusions

LDPE modified with mixture of raspberry, rosemary, and pomegranate CO_2_ extracts (aPE) obtained via cast extrusion were found to be a bacteriolytic against *S. aureus*. aPE also exhibited a low, antibacterial effect on the growth of *B. subtilis*, *E. coli*, and *P. syringae*. Due to the antibacterial activity of the obtained LDPE active film, the film could be used as a packaging material to extend the shelf-life of selected food products such as pork, beef, poultry, and processed meats, e.g., sausage. LDPE film with active agents incorporated into the polymer matrix was also found to be active against Φ6 particles, selected as coronavirus particles. The active film could be also effective against SARS-CoV-2 particles, and it could offer the possibility of decreasing the transmission of the virus from a packaging material to humans’ hands. The presence of a mixture of CO_2_ extracts in the polymer matrix affected its mechanical properties. It was observed that all parameters describing mechanical properties decreased due to weak miscibility of active agent with the polymer matrix. It seems to be a disadvantage from a mechanical point of view; however, weak bonding of additive in the matrix is required, due to migration of modifier onto the surface (confirmed with FTIR results) and increase of availability of active mixture for pathogens. The extracts incorporated into the polymer matrix did not affect phase transitions and thermal stability of polyethylene. Additionally, aPE exhibited barrier properties towards UV radiation, what is another functional feature, e.g., protection of food against aging. To summarize, the LDPE active film could be used as a packaging material to protect a food product against microbial spoilage on the internal side of packaging and to protect customers (hands) against bacteria and viruses on the outer layer of the packaging. This film may also be used to cover the surfaces that are often touched by human hands. This could limit the spread of coronavirus particles and bacterial strains transmitted by humans.

## Figures and Tables

**Table 1:** The physicochemical properties of pure PE (K) and PE films with an active mixture (aPE): tensile properties and color analysis.

Sample Acronym	Mechanical Properties				Color		
	TS (MPa)	EB (%)	YM (MPa)	Thickness (mm)	L*	a*	b*
K	18.2 (7.6 *)	564 (8.1 *)	127 (17.5 *)	0.14	96.9	0.06	0.18
aPE	13.6 (2.1 *)	427 (88.0 *)	110 (16.5 *)	0.20	96.4	−0.28	2.80

*—Standard deviation; “TS”—tensile strength; “EB”—elongation at break; “YM”—Young’s modulus.

## Data Availability

Data sharing is not applicable to this article.

## Supplementary Materials

The following are available online at https://www.mdpi.com/article/10.3390/ijms222413438/s1.
