# Supplementary material for: Polyethylene Films Containing Plant Extracts in the Polymer Matrix as Antibacterial and Antiviral Materials
Source: Int J Mol Sci. 2021 Dec 14;22(24):13438. doi: 10.3390/ijms222413438 (PMC8708998; doi:10.3390/ijms222413438)
Supplement: Supplementary file 1 [file ijms-22-13438-s001.zip › ijms-1494160-supplementary.pdf]

Suplementary Materials

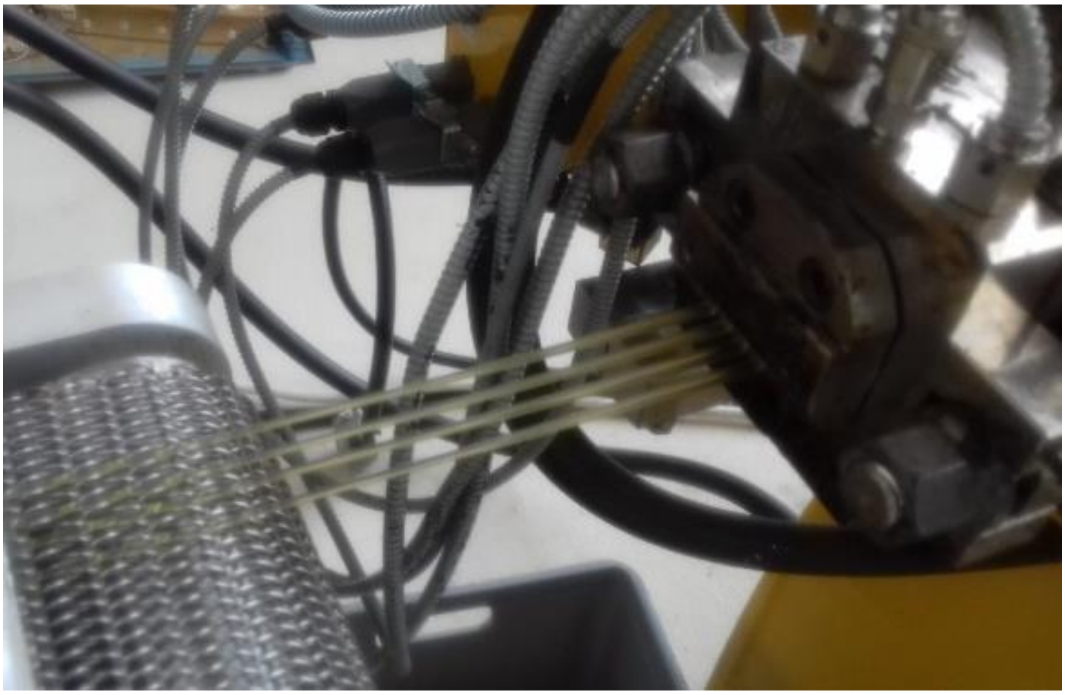

Figure S12 left: Regranulation of PE with the extracted mixture.

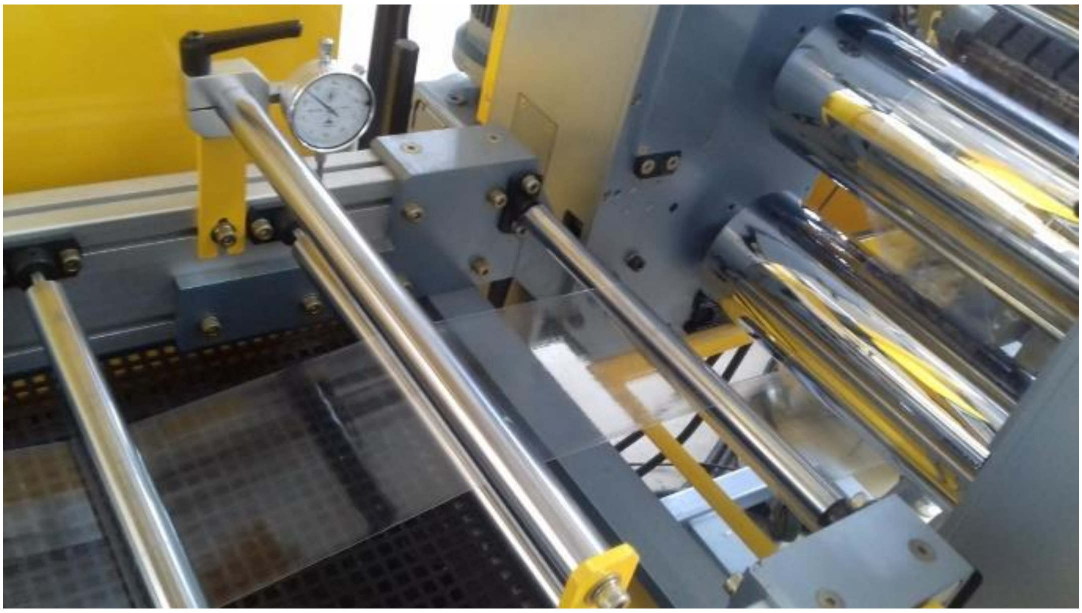

Right 12 right: Extrusion of the aPE.
